# User Interface and User Experience Testing of an Online Translation of the REACH II Intervention: GamePlan4Care

**DOI:** 10.1093/geroni/igab046.013

**Published:** 2021-12-17

**Authors:** Alan Stevens, Thomas Birchfield, Kira Swensen, Joseph Banda, Jinmyoung Cho

**Affiliations:** Baylor Scott & White Health Research Institute, Temple, Texas, United States

## Abstract

GamePlan4Care (GP4C) is a web-based adaptation of the Resources for Enhancing Alzheimer’s Caregiver Health II (REACH II) caregiver intervention, redesigned and reformatted for online delivery. The goal of GP4C is to create an online family caregiver support platform that facilitates self-directed exposure to evidence-based skills-training and support for dementia caregivers. This approach of utilizing technology enhanced with live support has the potential for scalability and sustainability. In preparation for an ongoing randomized clinical trial, the GP4C platform underwent industry standard user interface/user experience (UI/UX) testing with dementia caregivers as part of an iterative design process. Testing of caregiver’s reaction to technical and content-related aspects of the system was conducted with 31 caregivers. The thematic analysis revealed three themes for technical aspects (logical flow, suggestions on features, innovative resource) and two themes for content aspects (satisfaction and engagement). We will discuss technical and content modifications resulting from UI/UX.

